# Supplementary material for: Frequent disturbances enhanced the resilience of past human populations
Source: Nature. 2024 May 1;629(8013):837–42. doi: 10.1038/s41586-024-07354-8 (PMC11111401; doi:10.1038/s41586-024-07354-8)
Supplement: Supplementary file 1 — Supplementary Information [file 41586_2024_7354_MOESM1_ESM.docx]

**Supplementary Information for “Frequent disturbances enhanced the resilience of past human populations”**

**Authors:**

Philip Riris^1^*

Fabio Silva^1^

Enrico Crema^2^

Alessio Palmisano^3^

Erick Robinson^4,5,6^

Peter E. Siegel^7^

Jennifer C. French^8^

Erlend Kirkeng Jørgensen^9^

Shira Yoshi Maezumi^10^

Steinar Solheim^11^

Jennifer Bates^12^

Benjamin Davies^13^

Yongje Oh^12^

Xiaolin Ren^14^

1. Department of Archaeology & Anthropology, Bournemouth University, Poole, UK
2. Department of Archaeology, University of Cambridge, Cambridge, UK
3. Department of Historical Studies, University of Turin, Torino, Italy
4. Native Environment Solutions LLC, Boise, Idaho, USA
5. Division of Atmospheric Sciences, Desert Research Institute, Reno, Nevada, USA
6. School of Human Evolution and Social Change, Arizona State University, Tempe, Arizona, USA
7. Department of Anthropology, Montclair State University, Montclair, New Jersey, USA
8. Department of Archaeology, Classics, and Egyptology, University of Liverpool, Liverpool, UK
9. NIKU High North Department, Norwegian Institute for Cultural Heritage Research, Tromsø, Norway
10. Department of Archaeology, Max Planck Institute of Geoanthropology, Jena, Germany
11. The Museum of Cultural History, University of Oslo, Oslo, Norway
12. Department of Archaeology and Art History, Seoul National University, Seoul, Republic of Korea
13. Environmental Studies, Tufts University, Boston, Massachusetts, USA
14. Institute for the History of Natural Sciences, Chinese Academy of Sciences, Beijing, People’s Republic of China

*[priris@bournemouth.ac.uk](mailto:priris@bournemouth.ac.uk)

**Table of contents:**

Supplementary table legends S1-S18 2

Supplementary Methods 3

Supplementary references 4

**Supplementary Table Legends**

**Table S1**: **Summary of attributes of regions used in this study.** Locations, number of dates, the use of a null hypothesis significance testing approach in the original study, whether downturns are addressed in the original study, any subdivisions, overall timespan. Full table with bibliographic information at: <https://dx.doi.org/10.5281/zenodo.10061467>

**Table S2: Resistance and resilience metrics extracted from periods of population downturn detected in posterior predictive checks.** Full table (n = 169) in repository: <https://dx.doi.org/10.5281/zenodo.10061467>

**Table S3: Radiocarbon data for Arid Zone Australia.** Comma-separated value file (.csv) in UTF-8 encoding

**Table S4: Radiocarbon data for Caribbean Archipelago.** Comma-separated value file (.csv) in UTF-8 encoding

**Table S5: Radiocarbon data for China Central Plains.** Comma-separated value file (.csv) in UTF-8 encoding

**Table S6: Radiocarbon data for Circumpolar Norway.** Comma-separated value file (.csv) in UTF-8 encoding

**Table S7: Radiocarbon data for Greece.** Comma-separated value file (.csv) in UTF-8 encoding

**Table S8: Radiocarbon data for Highland and Coastal Peru.** Comma-separated value file (.csv) in UTF-8 encoding

**Table S9: Radiocarbon data for Italy, Sardinia, and Sicily.** Comma-separated value file (.csv) in UTF-8 encoding

**Table S10: Radiocarbon data for Korean Peninsula.** Comma-separated value file (.csv) in UTF-8 encoding

**Table S11: Radiocarbon data for Near East.** Comma-separated value file (.csv) in UTF-8 encoding

**Table S12: Radiocarbon data for Southeastern Norway.** Comma-separated value file (.csv) in UTF-8 encoding

**Table S13: Radiocarbon data for South Africa (GCFR).** Comma-separated value file (.csv) in UTF-8 encoding

**Table S14: Radiocarbon data for South Africa (SRZ).** Comma-separated value file (.csv) in UTF-8 encoding

**Table S15: Radiocarbon data for Tropical Lowlands.** Comma-separated value file (.csv) in UTF-8 encoding

**Table S16: Radiocarbon data for Utah.** Comma-separated value file (.csv) in UTF-8 encoding

**Table S17: Radiocarbon data for Wyoming.** Comma-separated value file (.csv) in UTF-8 encoding

**Table S18: Radiocarbon data for Yukon.** Comma-separated value file (.csv) in UTF-8 encoding

**Supplementary Methods**

The following Zenodo repository contains all the necessary material to reproduce the results reported in the text: <https://zenodo.org/doi/10.5281/zenodo.10061466>. At a high level, the file *resistance-resilience.RProj* can be opened within RStudio to access and run the entire workflow.

1. **Contents**

The Supplementary Methods is organised into six main folders:

**data** – Folder containing **Table S1**, a summary of attributes of regions used in this study. Locations, number of dates, the use of a null hypothesis significance testing approach in the original study, whether downturns are addressed in the original study, any subdivisions, overall timespan are reported. The **Table S2** collates all resilience metrics extracted from periods of population downturn detected in posterior predictive checks. Original radiocarbon date tables for 16 regions are in **Tables S3-18**.

**scripts** – Three R scripts for running Bayesian MCMC models, statistical modelling of results, and producing outputs.

**fits** & **output** – the results of running the above scripts.

**figures** & **Extended Data** – figures and tables produced for the main text and for the **Extended Data**.

1. **Data**

Raw data for the MCMC analysis can be found in the **data** folder, comprising 18 tables (.csv format) of archaeological radiocarbon dates with accompanying metadata (**Tables S1-18)**.

1. **Analysis**

***Bayesian MCMC***

Code for performing Bayesian Markov Chain Monte Carlo analysis on aggregated radiocarbon data (*mcmc.R*). Please note that, given the long processing time and memory requirements for each MCMC fit, the script contains code to reproduce a single example: Southeastern Norway. This is one of the smaller datasets (617 dates), and takes approximately ~6 hours to complete on an Intel(R) Core(TM) i5-9600 CPU @ 3.10GHz with 16 GB of DDR3 RAM. However, any of the 18 radiocarbon datasets can be substituted in this script and the parameters altered per Table S1 to obtain posteriors for any case study. The **output** folder contains the full results of the Bayesian MCMC analysis: MCMC diagnostics, parameters, posterior checks, and resistance-resilience metrics collected on each fit, including traceplots, Rhat, and ESS checks.

***Resistance-resilience metrics***

Code for the resmet() function is also contained in the *mcmc.R* file. resmet() is an adaptation of Edinborough et al.'s post-hoc statistical test for demographic events in written and oral history (https://doi.org/10.1073/pnas.1713012114). The inspiration for this function - p2pTest() in rcarbon - is for use with objects of class ‘SpdModelTest’. This function extends the principle to ‘spdppc’ objects.

Following Riris and De Souza (ref. 12), Nimmo et al. (ref. 52), Cantarello et al. (ref. 53), and Van Meerbeek et al. (ref. 11), this will perform post-hoc tests for resistance and resilience on marks of an ‘spdppc’ object over all periods where summed probability distributions (SPDs) are below growth model expectations ('downturns'). These two metrics are defined as the ability to absorb disturbances and "bounce back" following disturbances, respectively. They are normalised relative to the value of the SPD at the start of the interval of interest and fully described in the **Methods** section of the main text.

The function outputs a data frame containing the value of both metrics, as well as the duration, end- and start-times of downturns, and the time to SPD minimum, all in calendar years Before Present. Parameter 'LD' (short for lag/duration) is the Time to SPD minimum normalised by the downturn duration - which we term 'Pace' in the main text.

Raw results on individual posterior predictive checks can be found in the mcmc_metrics subfolder. *resistance-resilience_metrics.csv* contains the compiled, cleaned, and annotated dataset used in statistical modelling.

***Statistical Modelling***

Code for performing linear mixed-effect modelling on resistance-resilience metrics is contained in the *statisticalmodelling.R* file. It generates fitted models and diagnostics from the file *resistance-resilience_metrics.csv.*

1. **Display items**

Figures and tables for the main paper text and the Materials & Methods can be found in the relevant sub-folders. The *plotting.R* script produces **Figures 2-3** and **Extended Data Figures 1-7**.

**Supplementary references**

1. Nimmo, D.G., R. MacNally, S.C. Cunningham, A. Haslem, A.F. Bennett. Vive la résistance: reviving resistance for 21st century conservation. *TREE* **30**, 516-23 (2015). <https://doi.org/10.1016/j.tree.2015.07.008>
2. Cantarello, E., A.C. Newton, P.A. Martin, P.M. Evans, A. Gosal, M.S. Lucash. Quantifying resilience of multiple ecosystem services and biodiversity in a temperate forest landscape. *Ecol. Evol.* **7**, 9661-75. <https://doi.org/10.1002/ece3.3491>
