# Supplementary material for: Frequent disturbances enhanced the resilience of past human populations
Source: Nature. 2024 May 1;629(8013):837–42. doi: 10.1038/s41586-024-07354-8 (PMC11111401; doi:10.1038/s41586-024-07354-8)
Supplement: Supplementary file 2 — Supplementary Tables 1–18. [file 41586_2024_7354_MOESM2_ESM.pdf]

nature portfolio

Corresponding author(s): Philip Riris

Last updated by author(s): 14/03/2024

Reporting Summary

Nature Portfolio wishes to improve the reproducibility of the work that we publish. This form provides structure for consistency and transparency in reporting. For further information on Nature Portfolio policies, see our Editorial Policies and the Editorial Policy Checklist.

Statistics

For all statistical analyses, confirm that the following items are present in the figure legend, table legend, main text, or Methods section.

n/a	Confirmed	
☐	☒	The exact sample size (*n*) for each experimental group/condition, given as a discrete number and unit of measurement
☐	☒	A statement on whether measurements were taken from distinct samples or whether the same sample was measured repeatedly
☐	☒	The statistical test(s) used AND whether they are one- or two-sided<br>*Only common tests should be described solely by name; describe more complex techniques in the Methods section.*
☐	☒	A description of all covariates tested
☐	☒	A description of any assumptions or corrections, such as tests of normality and adjustment for multiple comparisons
☐	☒	A full description of the statistical parameters including central tendency (e.g. means) or other basic estimates (e.g. regression coefficient) AND variation (e.g. standard deviation) or associated estimates of uncertainty (e.g. confidence intervals)
☐	☒	For null hypothesis testing, the test statistic (e.g. *F*, *t*, *r*) with confidence intervals, effect sizes, degrees of freedom and *P* value noted<br>*Give P values as exact values whenever suitable.*
☐	☒	For Bayesian analysis, information on the choice of priors and Markov chain Monte Carlo settings
☐	☒	For hierarchical and complex designs, identification of the appropriate level for tests and full reporting of outcomes
☒	☐	Estimates of effect sizes (e.g. Cohen's *d*, Pearson's *r*), indicating how they were calculated

Our web collection on statistics for biologists contains articles on many of the points above.

Software and code

Policy information about availability of computer code

Data collection	No code was used.
Data analysis	The code supporting the findings of this study are available in Zenodo with the identifier doi: 10.5281/zenodo.10061467 (https://dx.doi.org/10.5281/zenodo.10061467).

For manuscripts utilizing custom algorithms or software that are central to the research but not yet described in published literature, software must be made available to editors and reviewers. We strongly encourage code deposition in a community repository (e.g. GitHub). See the Nature Portfolio guidelines for submitting code & software for further information.

Data

Policy information about availability of data

All manuscripts must include a data availability statement. This statement should provide the following information, where applicable:

- Accession codes, unique identifiers, or web links for publicly available datasets
- A description of any restrictions on data availability
- For clinical datasets or third party data, please ensure that the statement adheres to our policy

The data supporting the findings of this study are available in Zenodo with the identifier doi: 10.5281/zenodo.10061467 (https://dx.doi.org/10.5281/zenodo.10061467).

Research involving human participants, their data, or biological material

Policy information about studies with human participants or human data. See also policy information about sex, gender (identity/presentation), and sexual orientation and race, ethnicity and racism.

Reporting on sex and gender	NA
Reporting on race, ethnicity, or other socially relevant groupings	NA
Population characteristics	NA
Recruitment	NA
Ethics oversight	NA

Note that full information on the approval of the study protocol must also be provided in the manuscript.

Field-specific reporting

Please select the one below that is the best fit for your research. If you are not sure, read the appropriate sections before making your selection.

☐ Life sciences ☒ Behavioural & social sciences ☐ Ecological, evolutionary & environmental sciences

For a reference copy of the document with all sections, see nature.com/documents/nr-reporting-summary-flat.pdf

Behavioural & social sciences study design

All studies must disclose on these points even when the disclosure is negative.

Study description	Quantitative meta-analysis of archaeological radiocarbon data.
Research sample	Approx. 40,000 georeferenced radiocarbon dates over 16 global study locations collected from the extant research literature, which are representative of the spatio-temporal variability in land use, environment, social organisation, technology, political complexity, and extent of prehistoric human populations. In each study region, the number of radiocarbon dates are an accurate reflection of the total number of dates made on archaeological remains.
Sampling strategy	Studies were reviewed based on three criteria: evidence for significant downturns, their scope, and the inclusion of radiocarbon datasets. A lack of any single criterion resulted in the exclusion of a study. Cases with no reported downturns were not included, nor were those whose scope was restricted to specific activities within a regional radiocarbon dataset, such as flint mining. No sample size calculation was performed, however, our smallest regional sample size (n=272) is sufficient to replicate original study results and extract the features of population variability over time that are of interest to the study. All radiocarbon samples were included, where they reflect an archaeologically dated depositional event.
Data collection	We included all radiocarbon data reported by the authors of the original study. Where published data have been superseded by later compilations, dates were added from the People3k database, a systematic compilation of cleaned radiocarbon dates, based on the geographical area of the original study, and duplicates (based on laboratory code) were removed. Data were collected and collated with Microsoft Excel. The researcher was not blinded to experimental condition or the study hypothesis, insofar as this is applicable.
Timing	September 2021 - January 2022.
Data exclusions	No data were excluded.
Non-participation	Not applicable.
Randomization	Not applicable.

Reporting for specific materials, systems and methods

We require information from authors about some types of materials, experimental systems and methods used in many studies. Here, indicate whether each material, system or method listed is relevant to your study. If you are not sure if a list item applies to your research, read the appropriate section before selecting a response.

Materials & experimental systems

n/a	Involved in the study
☒	☐ Antibodies
☒	☐ Eukaryotic cell lines
☒	☐ Palaeontology and archaeology
☒	☐ Animals and other organisms
☒	☐ Clinical data
☒	☐ Dual use research of concern
☒	☐ Plants

Methods

n/a	Involved in the study
☒	☐ ChIP-seq
☒	☐ Flow cytometry
☒	☐ MRI-based neuroimaging

Plants

Seed stocks	NA
Novel plant genotypes	NA
Authentication	NA
